# Using peer education to improve diabetes management and outcomes in a low-income setting: a randomized controlled trial

**DOI:** 10.1186/s13063-019-3656-1

**Published:** 2019-09-02

**Authors:** Till Seuring, Marthoenis, Sabrina Rhode, Lisa Rogge, Holger Rau, Stéphane Besançon, Hendra Zufry, Hizir Sofyan, Sebastian Vollmer

**Affiliations:** 1 Leibniz Institute for Prevention Research and Epidemiology - BIPS GmbH, Achterstr. 30, 28359 Bremen, Germany; 2 Jl Tgk Tanoh Abee, Darussalam, Banda Aceh, Prodi Magister Keperawatan Unsyiah, Syiah Kuala University, Banda Aceh, Aceh 23111 Indonesia; 3 Heidelberg Institute of Global Health, Heidelberg University, Im Neuenheimer Feld 130.3, 69120 Heidelberg, Germany; 4 Leibniz University of Hannover, Königsworther Platz 1, 30167 Hannover, Germany; 5 University of Göttingen, Platz der Göttinger Sieben 3, 37073 Göttingen, Germany; 6 Santé Diabète, 17 avenue Malherbe, 38100 Grenoble, France; 7 Zoeinal Abidin Hospital, Jl Daud Beureueh, Banda Aceh, Aceh 23000 Indonesia; 8 Jalan Teuku Nyak Arief, Darussalam, Kopelma Darussalam, Syiah Kuala University, Banda Aceh, Aceh 23111 Indonesia; 9 Centre for Modern Indian Studies (CeMIS), University of Göttingen, Waldweg 26, Altbau, 37073 Göttingen, Germany

**Keywords:** Peer education, Patient-led education, Diabetes, Cluster randomized controlled trial, Non-communicable diseases, Indonesia, Cost-effectiveness

## Abstract

**Background:**

Diabetes is an important health burden in Indonesia. However, diabetes management and treatment remain poor, with most people with diabetes in Indonesia not achieving the recommended blood glucose levels. Peer education may have particular potential in low-income settings in complementing diabetes care without being a large additional strain on the health system.

**Methods/design:**

This cluster randomized controlled trial aims to identify the effect of the implementation of peer education for patients with type 2 diabetes on diabetes-related outcomes in Aceh, Indonesia, which will complement the diabetes treatment provided at primary-care health posts (*puskesmas*). Altogether, 29 *puskesmas* were recruited in Banda Aceh and Aceh Besar, each of which was randomly assigned to either the control or the intervention group. Then, 534 people with diabetes were identified and recruited through their respective *puskesmas*. The intervention consists of up to two peer education groups per *puskesmas*, which are led by previously trained people with diabetes. Peer education sessions are held every month for 18 months, with follow-up data being collected 9 and 18 months after the first peer education session.

The main objective is to improve diabetes management and the health behavior of participants receiving peer education to reduce their average blood glucose levels as measured by glycated hemoglobin (HbA1c) levels. Secondary outcomes are the effects of peer education on lipid levels, waist circumference, blood pressure, quality of life, treatment adherence, diabetes knowledge, physical activity, and dietary diversity. Data sources for the measurement of outcomes include patient and health facility surveys and biomarker measurements. An economic evaluation will be conducted to assess the cost-effectiveness of the intervention.

**Discussion:**

This trial will contribute to the evidence on the effectiveness and cost-effectiveness of peer education in improving diabetes management in a low-income setting in Indonesia and in other comparable contexts.

**Trial registration:**

ISRCTN registry, ISRCTN68253014. Registered on 18 February 2019.

**Electronic supplementary material:**

The online version of this article (10.1186/s13063-019-3656-1) contains supplementary material, which is available to authorized users.

## Background

Public health systems in low- and middle-income countries are overburdened with the demands of delivering care to those with communicable and non-communicable diseases. People with the latter in particular often require long-term monitoring and support to achieve good health [1]. For diabetes, inadequate care or no access to care can lead to high rates of uncontrolled diabetes and complications. It is unclear, however, how better care can be provided in an environment with few resources, as is the case in low- and middle-income countries.

In Indonesia, diabetes has become one of the main contributors to the burden of disease, surpassing many communicable diseases, especially among adults [2]. However, diabetes treatment at the main public primary-care facilities, *puskesmas*, remains poor, which is partly due to the limited knowledge of health-care professionals about diabetes [2, 3]. Consequently, recent studies on diabetes in Indonesia indicate poor levels of control, with around 70% of patients having glycated hemoglobin (HbA1c) levels above 7% [4].

A potential way to complement the diabetes care available in Indonesia is the use of peer education, in which highly motivated and trained diabetes patients educate and support other patients with diabetes to manage the disease in daily life in a culturally appropriate way. Due to the need for patients to deal with their diabetes every day, and the potential of lifestyle changes and preventative behavior to prevent major diabetes complications, empowering patients by increasing their knowledge of diabetes care could theoretically yield positive results. Peer education focuses and relies on people with diabetes, rather than medical professionals, to improve diabetes care. Therefore, it may be less resource intensive than traditional approaches and particularly attractive in environments where access to and the quality of diabetes health care are very limited [5].

Several randomized controlled trials have been conducted in recent years to test the efficacy of peer education in improving diabetes outcomes in high- and in some low- and middle-income countries. Two systematic reviews have indicated that peer education overall can lead to statistically significant reductions in HbA1c levels [5, 6]. It appears that it is especially effective for some sub-groups of diabetes patients, such as those with relatively uncontrolled diabetes [5] and minorities [6]. Because most of the trials reviewed took place in high-income countries, these results may not be directly applicable to relatively poorer countries. The two studies that evaluated the effects of peer education in a middle-income country (China and Argentina), took place in an urban environment (La Plata, Argentina [7] and Hong Kong [8]).

Hence, there is a lack of evidence on the causal effect of peer education in low- and middle-income countries, in particular on its adaptability to non-urban environments where diabetes care mainly happens at the primary-care level.

Our study results may, therefore, play an important part in advancing the knowledge base on the treatment of diabetes in low- and middle-income countries. In particular, it may provide evidence of its effectiveness in a setting that consists of both urban and relatively remote rural areas, and where traditional beliefs around diabetes and the use of alternative medicines are still relatively common [4].

Moreover, our study will look at the effectiveness of peer education over a relatively long time of 18 months. It further aims not only to look at HbA1c, but also at changes in lipid levels as well as blood pressure and waist circumference, given their role as risk factors for diabetes-related complications. Because peer education relies on the ability of peer educators to train their peers and the peers’ ability to use this information to change their behavior, we will also assess if the personal characteristics of study participants can mediate the success of the intervention. In particular, we want to investigate the role of time and risk preferences, which may mediate the ability of participant to prioritize behavior change today to prevent diabetes complications in the future. Finally, our study will provide estimates of the cost-effectiveness of peer education in a low-income context of a developing country. This will add further evidence about the viability of peer education in health systems of low- and middle-income countries in terms of its financial costs and effectiveness.

## Methods/design

### Study setting

In Indonesia, most people with diabetes receive their regular diabetes treatment at the primary-care level at public health posts called *puskesmas*. These are government-mandated primary-care providers and the first point of contact for people seeking care in the public health system in Indonesia. Each *puskesmas* normally serves one sub-district, which has a population of 30,000 to 50,000. The main role of *puskesmas* is the delivery of primary outpatient care, but they are also used to promote and realize public health measures such as immunization, nutrition education and health information campaigns. About one-third of *puskesmas* in Indonesia provide basic inpatient care for emergency obstetric and neonatal care. Outpatient care efforts have mainly focused on communicable diseases, so that many *puskesmas*, especially in rural areas, provide a low quality of diabetes services [2]. This is characterized by a limited capacity to detect diabetes via diagnostic tests, and to treat and manage diabetes and its complications. Diabetes care is mainly limited to the use of medication to control the disease, and mostly disregards patient education on topics such as diet or exercise [2, 3]. The diabetes burden in Aceh is mostly unknown, but judging from national data, substantial [2]. This study is taking place at *puskesmas* in the districts Banda Aceh and Aceh Besar in the north of Sumatra, with Banda Aceh consisting of mostly urban areas and Aceh Besar of rural areas.

### Study design

The study is a cluster randomized controlled trial with a parallel group design, with the clusters consisting of *puskesmas* from Banda Aceh and Aceh Besar Regency.^1^ Peer education groups were randomly established in 50% of *puskesmas* after they joined the study and baseline data collection had been terminated. This study design allows us to establish two groups of participants (treatment and control group) so that we can causally identify the effect of the peer education on the primary and secondary outcomes. Blinding was possible at baseline, which preceded the allocation of clusters into treatment and control groups. However, after the randomization and allocation, the blinding of the treatment allocation of participants, project managers, and investigators is no longer possible. For the duration of the study, the control group will not receive any intervention beyond being informed about their blood test results. The Standard Protocol Items: Recommendations for Interventional Trials (SPIRIT) checklist is provided as an Additional file 1.

### Intervention: peer education

Peer support has been defined as “support from a person who possesses experiential knowledge of a specific behavior or stressor and similar characteristics as the target population” [9]. It has been shown to help reduce or prevent problematic health behaviors and alleviate vascular disease, HIV, and Parkinson’s disease among others [5]. The appeal of peer support is its ability to create nonhierarchical reciprocal relationships through the sharing of similar life experiences between the peer educator and the peers. Additionally, because peer education relies on non-professionals to improve the health outcomes of patients, it may be significantly less resource intensive than trying to achieve the same effects with professional health workers.

The intervention was designed in cooperation with the local expert team as well as experts experienced with the implementation of peer education in a low-income context in Mali [10]. Furthermore, qualitative interviews and focus group discussions with nurses working with diabetes patients at *puskesmas* informed the intervention design, in particular regarding practicable ways to train peer educators and to provide them with the means to transfer their knowledge successfully to their peer groups.

### Training of peer educators

Selected peer educators will receive training before and after the start of the implementation. A 2-day intensive training session by local physicians on diabetes and nutrition was carried out at the beginning of April 2019 before the start of the peer education sessions. It provided general information about diabetes as a disease, its risks, and the ways to treat it. This initial information session will be followed up by monthly half-day training sessions for the peer educators until the end of the study. These training sessions for peer educators will be led by two specially trained nurses, who, prior to each training session, will be educated by a member of the research team on the specific topic to be discussed. The topics and structure of the peer educator training sessions will be guided by the peer leader manual published by the International Diabetes Federation [11]. The goals of these additional education sessions [1] are to distribute the burden of training sessions for peer educators over a longer period of time [2], to maintain the motivation and commitment of peer educators over time [3], and to use feedback from the peer educators after their peer education sessions to adapt the training to the needs of the peer educators and their peers.

### Use of peer education

Depending on the number of patients recruited per *puskesmas* and the number of potential peer educators, one or two peer educators will be selected per *puskesmas*, to limit the group size to 13 participants. Peer education sessions are planned to be held once a month for 18 months. They will be conducted by the peer educator only, without the presence of a trained nurse or a member of the research staff, to avoid any potential changes in the behavior of the peer educator or the patients. To preserve some flexibility in how peer educators conduct the peer education sessions and react to the needs of the group, we will refrain from closely monitoring each session. Rather, we will use feedback from the peer educators about the sessions to determine if they are successful in discussing the planned topics.

### Research questions

The specific research questions are:

1. Will HbA1c levels decrease in patients who take part in peer education groups and if so, by how much?
2. What effect will peer education have on lipid levels, waist circumference, blood pressure, and quality of life?
3. What effect will peer education have on health and self-care behavior of participants in the intervention group?
4. Is the intervention cost-effectiveness?
5. What are important mediators for the effectiveness of peer education?

### Outcome measures

The primary outcome is the change in HbA1c levels from baseline to the final assessment. HbA1c will be collected at baseline, midline and the final assessment using point-of-care testing devices allowing for the immediate measurement of HbA1c levels. Secondary outcomes are:

- changes in lipids (total cholesterol, high-density lipoprotein, and triglycerides) collected using point-of-care testing devices at baseline, during the trial, and at the final assessment
- blood pressure
- waist circumference
- diabetes knowledge
- medication adherence (five-item Medication Adherence Scale, MARS-5 [12])
- diabetes distress (Diabetes Distress Scale 2 [13])
- healthy behaviors, such as smoking status and number of cigarettes per day, and physical activity levels (WHO global physical activity questionnaire [14])
- dietary diversity (dietary diversity questionnaire published by the Food and Agriculture Organization of the U.N. [15])

Finally, to assess the cost-effectiveness of the intervention, changes in health-care costs and changes in quality-adjusted life years (based on EQ-5D-3 L questionnaire [16]) will be used to calculate the incremental cost-effectiveness ratio of the intervention [16].

Regarding potential mediators, we specifically focus on the behavioral characteristics of the participants. In particular, we measure risk and time preferences as well as trust in other people using unincentivised questions. We also use the Collective Self-Esteem Scale, which measures the ability of participants to function in and identify with social groups [17], and the 13-item Self-Control Scale [18].

### Study duration

**Fig. 1:**
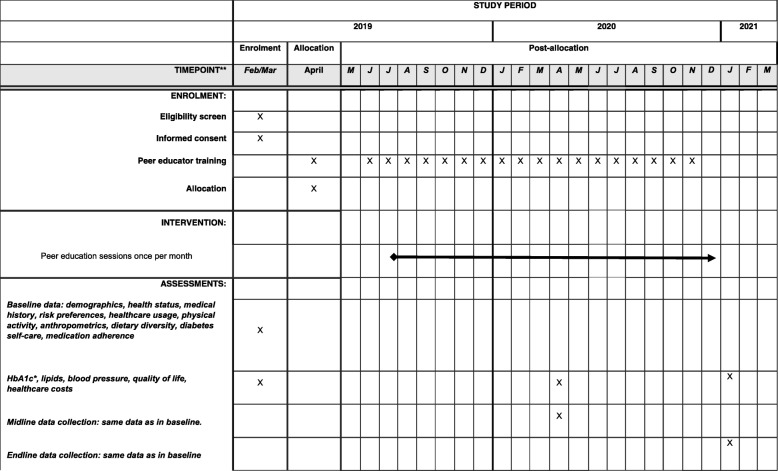
Schedule of enrolment, intervention, and assessments. HbA1c glycated hemoglobin

A qualitative study to inform the intervention was carried out in May and June 2018. As shown in the SPIRIT figure (Fig. 1), recruitment occurred in February and March 2019. Baseline data were collected in April 2019, including primary and secondary outcome variables, for all participating patients. The intensive training sessions of the selected peer educators took place in April 2019 and the peer groups were established in July 2019. The first follow-up data will be collected in April 2020, including primary and secondary outcome variables. This will be followed by an analysis of the preliminary results. The final assessment will be conducted in January 2021, including primary and secondary outcome variables. This will be followed by an analysis of the results and the establishment of peer support groups in the control group if the intervention proves to be effective.

### Number of participants and power calculation

The study aimed to include patients from all *puskesmas* in Banda Aceh and Aceh Besar.^2^ Power calculations indicated that with 680 participants in 34 clusters, an assumed intra-cluster correlation of HbA1c of 0.37, and a pooled standard deviation of HbA1c of 1.25, we would have 80% power to detect a difference of 0.5 in mean HbA1c using a significance level of *p* = 0.05 and 90% power to detect a difference of 0.686 using a significance level of *p* = 0.01. We chose a difference of 0.5 in mean HbA1c, as this has been determined as a clinically meaningful threshold [19]. At the end of the recruitment phase, we had been able to recruit 534 participants from 31 *puskesmas*. However, two *puskesmas* could not be used as separate cluster units since their low recruitment numbers prevented the formation of a peer education group. We decided to assign the participants from these *puskesmas* to the geographically closest *puskesmas*. This reduced the number of clusters from 31 to 29, which gives 80% power to detect a difference of 0.57 in mean HbA1c using a significance level of *p* = 0.05 and 90% power to detect a difference of 0.78 using a significance level of *p* = 0.01.

### Inclusion criteria

While previous studies have shown that there is a real benefit of peer education for people with very elevated HbA1c levels, we aim to include any person with type 2 diabetes. There are two main reasons for this decision. First, many *puskesmas* in Indonesia are currently not equipped to test HbA1c levels and would, hence, be unable to select patients based on their HbA1c levels. We, therefore, think that our intervention should reflect the reality of the current health system and investigate the suitability of measuring HbA1c levels for later implementation. Second, we suspect that there is also a benefit of joining peer education for those with better HbA1c levels, by helping them to prevent a worsening of the disease over time.

Therefore, we used the following inclusion criteria for patients in the peer education groups or the control groups:

- Patients treated in *puskesmas* for type 2 diabetes in the intervention area
- Patients who agreed to undergo the whole process of peer education
- Patients who agreed to carry out all biological and survey assessments as required by the protocol
- Patients aged 20–79 years
- Patients not enrolled in another research program

The peer educators will need to fulfill the following requirements:

- Can commit to attending 20 h of training
- Are willing to organize activities with other patients every month
- Have basic diabetes self-management knowledge and supportive non-judgmental communication skills
- Willing to lead
- Literate

### Exclusion criteria

- Pregnancy
- Being unable to physically attend peer education sessions

### Study procedures

The project has several phases. First, to inform the intervention design, qualitative interviews with nurses and diabetes patients were carried out. Then, for each *puskesmas*, patients with diabetes were recruited into the peer education program. An interview was carried out in each facility to receive the facility head’s consent to participate, to gather general information and information specific to diabetes treatment and, if possible, to obtain a list of diabetes patients. Additionally, health workers in the *puskesmas* and in the villages as well as patients who had already been interviewed were asked to suggest further people with diabetes who were associated with the relevant *puskesmas*.

At baseline, the patients recruited were interviewed by trained enumerators using a questionnaire. They were invited to the *puskesmas* on one predetermined day, during which the trained enumerators used point-of-care devices to test their HbA1c and lipid levels. The participants were informed of their test results. Blood was then drawn from the arm of participants by health-care professionals, which was used for laboratory as well as point-of-care tests of HbA1c and lipid profiles to determine the accuracy of the point-of-care test devices in the study setting under field conditions. In the middle and final assessments, only point-of-care tests using venous blood will be carried out.

In choosing potential peer educators, all participants were asked during the interviews if they would be interested in serving as a peer educator. Further, health facility staff were asked during the facility interview to suggest patients for this role. Out of the participants recruited, we determined potential peer educators based on three criteria: (1) their willingness to take on this role, (2) a recommendation from the health facility staff, and (3) how well they were already controlling their diabetes based on the HbA1c level from the baseline data.

Following data collection, the *puskesmas* were randomized. Because recruitment took place before randomization, the researchers, participants, and facility managers did not know which facilities would be allocated to the treatment and control groups. We used covariate-constrained randomization to ensure the treatment and control arms were balanced in terms of baseline covariates while maintaining the randomness of the allocation [20, 21]. The covariates took into account the composition of the group with regards to size of the group, and the age, sex, education, participation in other health programs,^3^ and diabetes control (mean HbA1c values) of the members, as well as the location of the *puskesmas* (rural or urban). Randomization was carried out using the statistics program Stata.

Peer education groups were formed at each *puskesmas* in the treatment group. Peer educators will receive continuous training in diabetes management skills and will be supported in the administrative processes (e.g., room booking) required to run their peer education group in their local sub-district.

To ensure that the effect of using peer education can be observed in comparison to normal patient education efforts, the control group will not receive any additional education apart from standard therapy.

As detailed in Fig. 1, 9 months after the first peer education session, a first follow-up will assess the implementation and the effects of peer education. The final assessment will take place 18 months after the first peer education session to assess the long-term effects of the intervention and the experience of participants.

We will then compare the outcomes between treatment and control groups, carry out a cost-effectiveness analysis, present the results to local health authorities, and discuss how to implement the program more widely if it proves to be effective.

### Economic evaluation

Costs related to the implementation of peer education will be collected from the the documented project expenditures. Data on resource use for diabetes treatment and care, including equipment, drugs, and doctor and hospital visits, will be collected retrospectively using cost information from the health insurance provider if possible, or via the expert opinion of local specialists. These data will be supplemented with information on participants’ health-care seeking behavior and utilization, drug use, and out-of-pocket payments collected through the surveys.

To estimate cost-effectiveness, the incremental cost-effectiveness ratio will be calculated from the health-care system perspective and the societal perspective taking into account out-of-pocket expenditures and costs related to participating in peer education. The incremental cost-effectiveness ratio is defined by the difference in costs between receiving no intervention plus standard diabetes care and the standard care plus intervention costs, divided by the difference in their effect. Here it represents the average incremental cost for peer education associated with one additional quality-adjusted life year.

### Data management

Data will be collected using tablets and directly entered into the electronic data entry program Open Data Kit (ODK collect). Data will be anonymized, and the original data will be stored separately on an encrypted hard storage device. Any biomarker data will be stored separately from the participant’s name on an encrypted hard storage device. The results of the biomarker tests will be reported back to the participants.

### Statistical methods

Random sampling and random assignment of intervention units to the treatment and control groups will allow us to interpret differences in mean outcomes as causal effects of the intervention. By comparing outcomes between participants in the peer education groups compared to study participants in the control group, we will be able to draw conclusions of the intent-to-treat effect and the size of the effect.

Because covariate-constrained randomization was used to balance the baseline characteristics, it is necessary to account for this design at the analysis stage [21, 22]. We will use general (generalized) linear mixed models to incorporate data structures that are both hierarchical and longitudinal and adjust for the covariates used during the covariate-constrained randomization [21, 22]. Standard errors will be clustered at the intervention unit level (*puskesmas*).

### Data monitoring

Because this study does not involve any testing of medications or procedures that are outside the usual care provided to individuals with type 2 diabetes, monitoring patient safety will be limited to mandatory reporting of adverse events and unanticipated problems. Participants will be made aware of unexpected test findings identified during the trial. Adverse events include clinical reactions to blood draws related to the study, as well as any reported stigma or physical or mental harm as a result of participation in the study, including disclosure of diabetes status.

### Foreseeable risks, discomforts, and inconvenience to participants

Trial participation is voluntary, so that participants may leave the trial at any time for whatever reason. Because the provision of education should only add to the participants’ knowledge about how to manage their diabetes, we see no immediate risks for participants. This additional information may lead to an increased awareness of the risks of sustained high glucose levels and could increase anxiety in participants. Theoretically, the personal experience or beliefs of the peer educators may lead them to diverge from the educational material to promote potentially harmful or ineffective practices for treating diabetes.

### Provisions in place to minimize risk

The possible high returns from participating in the peer education sessions and from knowing their HbA1c and other biomarkers will be emphasized to motivate patients to continue trial participation. Because the control and intervention groups will receive their blood test results, any effects on our outcome variables due to receiving this information should not affect the estimate of the intervention effect. To prevent peer educators from promoting ineffective and potentially harmful treatments of diabetes, we will carefully select peer educators and emphasize the need to follow the content of the education materials during the peer education sessions. We may also aim to address some myths surrounding diabetes and how diabetes should be treated to increase awareness of the potential dangers of ineffective treatments, which peer educators can use to address such beliefs in their peer education sessions.

## Discussion

The goal of this cluster randomized controlled trial is to test the effectiveness of peer education to improve diabetes-related outcomes in a low-income setting with a high burden of diabetes.

This trial has several advantages over previous trials carried out on peer education for diabetes in low- and middle-income countries. First, its relatively long follow-up allows us to observe the effectiveness of peer education over a longer time. Second, because we measure not only HbA1c but also blood lipids, we will be also able to investigate the effects of peer education on these risk factors for diabetes complications. Further, we collect information on risk and time preferences, which will allow us to assess whether the effectiveness of the intervention depends on such personal patient characteristics. Finally, we will provide a first estimate of the cost-effectiveness of peer education in a low-income context with the goal of informing policy makers of the costs and benefits of the intervention, and in comparison to other interventions in the health sector.

If the trial shows that peer education is effective in improving diabetes outcomes in Indonesia, the next step will be to explore the potential for the intervention to be integrated into the local health-care system, considering the practicability of the intervention and its cost-effectiveness. We have been in contact with local health authorities who are supporting the study and will disseminate the results to them and other authorities once the study has concluded.

### Trial status

The current version of this protocol is 1.5, dated 5 August 2019. Enrollment began on 18 February 2019 and concluded on 10 April 2019.

## Additional files

Additional file 1: SPIRIT 2013 Checklist. Recommended items to address in a clinical trial protocol and related documents. (DOC 122 kb)

Additional file 2: Consent Forms (patients and facilites) in Indonesian. (PDF 399 kb)

Additional file 3: Consent Form (patients and facilities) in English. (PDF 313 kb)

## Data Availability

The data and materials will be made available to the research and practice community on written request after the data set has been cleaned and locked for analysis, and once the trial has been concluded for at least 1 year and the primary results have been published. The data will be anonymized before sharing by removing any identifying information and stored in a CSV file to allow importation into multiple software packages. The data will be made available for non-profit scientific research purposes. Consent for data sharing with other researchers has been obtained from the participants. Interested researchers will need to provide us with their name, affiliation, and the goal of their research project for which they want to use the data. The data will then be transferred electronically in encrypted password-protected files with the password sent separately.

## Abbreviations

HbA1c: Glycated hemoglobin

## Footnotes

1 A regency is an administrative unit in Indonesia comparable to municipalities, just below the level of provinces. Like cities (e.g., Banda Aceh), regencies have their own local governments and parliaments.

2 There are 39 *puskesmas* in total, of which seven were excluded ex ante for practical reasons as they are located either in the remote mountainous area or on an island, so that it would not have been feasible to conduct either the survey or the training of the peer educators with the given resources.

3 Namely, either the Prolanis or the Posbindu program, which are two *puskesmas*-based national programs targeted at better preventing and managing diabetes and hypertension.
