# Supplementary material for: Using peer education to improve diabetes management and outcomes in a low-income setting: a randomized controlled trial
Source: Trials. 2019 Sep 2;20:548. doi: 10.1186/s13063-019-3656-1 (PMC6719346; doi:10.1186/s13063-019-3656-1)
Supplement: Supplementary file 2 — Consent Forms (patients and facilites) in Indonesian. (PDF 399 kb) [file 13063_2019_3656_MOESM2_ESM.pdf]

A: FORMULIR PERSETUJUAN INSTITUSI KESEHATAN

<table>
<tr><th colspan="2">Informasi Kegiatan</th></tr>
<tr><td>Peneliti Utama: Prof Dr Sebastian Vollmer</td><td>Institusi : Georg-August University of Goettingen</td></tr>
<tr><td>Alamat :
Georg-August Universität
Goettingen
Platz der Goettinger Sieben 3
37073 Goettingen
Jerman</td><td>Till Seuring
Phone: +49 (0) 421 – 21 85 69 23
Email: till.seuring@gmail.com
Marthoenis Marthoenis
Phone: +62 (0) 812 – 63 46 34 49
Email: marthoenis@unsyiah.ac.id</td></tr>
<tr><td>Anggota Peneliti :
Dr. Till Seuring
Dr.Marthoenis Marthoenis</td><td>Institusi:
Leibniz Institute for Prevention Research and Epidemiology - BIPS
Universitas Syiah Kuala</td></tr>
</table>

Nama saya ____________________ Saat ini saya bekerja dengan tim dari Universitas di Goettingen, Jerman dan Universitas Syiah Kuala, Indonesia. Kami sedang melakukan intervensi tentang penggunaan pendidikan sebaya bagi penderita diabetes untuk melengkapi perawatan diabetes di Puskesmas. Meskipun penelitian yang kami lakukan adalah bersifat independen namun dari pemerintah, Dinas Kesehatan Kabupaten Aceh mendukung kegiatan ini dan Komite Izin Etika di Fakultas Kedokteran Universitas Syiah Kuala telah memberikan persetujuan untuk melakukan penelitian ini [tunjukkan surat dukungan]....

Pendidikan sebaya adalah intervensi yang bertujuan untuk meningkatkan pengetahuan tentang perawatan diri diabetes dan perilaku sehat pasien dengan diabetes tipe 2, untuk meningkatkan kadar glukosa darah dan mengurangi faktor risiko diabetes seperti obesitas, kelebihan berat badan dan tekanan darah tinggi. Pendidikan sebaya telah sukses dilakukan sesuai dengan tujuan tersebut di negara lain, sehingga diharapkan dapat membantu mengurangi beban diabetes di Aceh juga. Untuk melaksanakan proyek ini kami akan memilih pasien dengan diabetes tipe 2 serta pendidik sebaya, yaitu penderita diabetes yang akan menerima pelatihan tambahan dalam pendidikan diabetes. Pendidik

sebaya ini kemudian akan memimpin kelompok orang dengan diabetes tipe 2 dan mendidik mereka tentang cara mengobati diabetes mereka dengan lebih baik dan apa yang harus dilakukan untuk mengubah gaya hidup mereka.

Tujuan dari penelitian ini adalah bahwa pada akhirnya, kelompok pendidikan sebaya akan dibentuk di semua fasilitas kesehatan masyarakat yang berpartisipasi. Tetapi karena keterbatasan anggaran, kami tidak dapat memperkenalkan kelompok-kelompok tersebut dengan pelatihan gratis di semua fasilitas pada saat yang bersamaan. Oleh karena itu, di awal kami akan mulai menerapkannya dengan menggunakan 50% dari pasien sebuah fasilitas kesehatan dan 50% lainnya akan mendapatkannya dalam paruh kedua di tahun 2019. Ini akan ditentukan secara acak oleh institusi akan mendapatkan intervensi pertama. Saat ini kami mengunjungi semua fasilitas kesehatan untuk meminta persetujuan mereka untuk mengambil bagian dalam studi penelitian. Pada pertengahan 2018, seseorang dari tim kami akan kembali ke institusi kesehatan Anda dan memberi tahu Anda apakah fasilitas Anda dipilih untuk pelaksanaannya pada pertengahan 2018 atau di paruh kedua tahun 2019.

Penelitian kami mungkin tidak mengubah hal-hal penting dalam waktu yang cepat, karena itu hal ini akan bergantung pada pemerintah lokal dan nasional. Kami di sini untuk belajar dari Anda dan juga dari pasien Anda, dan kami tidak dapat berjanji untuk memperbaiki keadaan secara sempurna. Partisipasi dalam penelitian ini bersifat sukarela. Namun, sangat diharapkan partisipasi Anda dalam survei ini karena itu menjadi hal penting untuk membantu kami belajar tentang cara mengurangi beban diabetes di provinsi Aceh dan di seluruh Indonesia.

Jika Anda memiliki pertanyaan lebih lanjut, Anda dapat menghubungi:

Tim Peneliti dari Jerman
Till Seuring
Email: t.seuring@gmail.com
Phone: +49 (0) 421 – 21 85 69 23
Mobile: +49 (0) 176 – 47 13 41 74

Tim Peneliti dari Indonesia
Marthoenis
Email: marthoenis@unsyiah.ac.id
Phone: +62 (0) 812 – 63 46 34 49

Apakah Anda memiliki pertanyaan pada saat ini? Anda dapat menanyakan apa pun yang tidak Anda mengerti atau apa pun yang ingin Anda ketahui.

Apakah Anda ingin berpartisipasi?

LEMBAR PERSETUJUAN

**KESEDIAAN MEMBERIKAN INFORMASI**		
II 1	Nama Lembaga Kesehatan	Nama:

Saya, _________________________________, setelah diinformasikan tentang semua aspek penelitian ini, dan setelah semua pertanyaan dan kekhawatiran saya tentang proyek ini terjawab, saya secara sukarela menerima untuk berpartisipasi dalam penelitian ini di fasilitas kesehatan saya. Saya berkomitmen dan fasilitas kesehatan saya untuk mendukung prosedur yang dijelaskan di atas. Saya memiliki kesempatan untuk mengajukan pertanyaan apa pun terkait penelitian tersebut. Saya memahami prosedur proyek dan bagaimana informasi akan diperlakukan secara rahasia, tanpa mengungkapkan identitas orang yang berpartisipasi dalam penelitian dalam hasil apa pun yang dilaporkan atau dipublikasikan. Saya memberikan wewenang untuk memberikan akses pada informasi yang saya berikan kepada semua anggota tim peneliti, karena mengetahui bahwa informasi ini akan digunakan secara rahasia. Saya memahami bahwa persetujuan saya ini dan tidak mengambil hak hukum apa pun dalam hal kelalaian atau kesalahan hukum lainnya dari siapa pun yang terlibat dalam penelitian ini. Lebih lanjut saya memahami bahwa tidak ada dalam formulir persetujuan ini yang dimaksudkan untuk berlawanan dengan hokum negara dan norma sosial di daerah yang berlaku.

Nama Ketua Lembaga (*print/tulis tangan*):

Tanda tangan Ketua:

Tanggal:

Nama Enumerator (*print/tulis tangan*):

Tanda tangan Enumerator:

Tanggal:

B: FORMULIR PERSETUJUAN DARI TENAGA KESEHATAN

**Informasi Kegiatan**	
Peneliti Utama: Prof Dr Sebastian Vollmer	Institusi : Georg-August University of Goettingen
Alamat :<br><br>Georg-August Universität<br><br>Goettingen<br><br>Platz der Goettinger Sieben 3<br><br>37073 Goettingen<br><br>Jerman	Till Seuring<br><br>Phone: +49 (0) 421 – 21 85 69 23<br><br>Email: till.seuring@gmail.com<br><br>Marthoenis Marthoenis<br><br>Phone: +62 (0) 812 – 63 46 34 49<br><br>Email: marthoenis@unsyiah.ac.id
Anggota Peneliti :<br><br>Dr. Till Seuring<br><br>Dr.Marthoenis Marthoenis	Institusi: Leibniz Institute for Prevention Research and Epidemiology - BIPS<br><br>Institusi: Universitas Syiah Kuala

Catatan Penting untuk Enumerator: Tidak ada staf peneliti yang harus menekan, memaksa atau menipu responden dalam upaya untuk memastikan partisipasi mereka. Staf juga harus mencoba memastikan bahwa responden tidak ditekan oleh tenaga kesehatan lain atau oleh kepala fasilitas kesehatan. Staf tidak boleh membuat janji apa pun yang tidak bisa atau tidak mungkin mereka tepati. Para responden akan bebas untuk menarik diri dari penelitian ini kapan saja. Sementara prosedur penelitian dirancang untuk memastikan bahwa persetujuan telah diinformasikan dan bersifat sukarela, satu-satunya orang yang benar-benar dapat memastikannya adalah Enumerator sendiri. Anda harus berusaha semaksimal mungkin untuk memastikan peserta memahami maksud penelitian ini dan merasa bebas untuk tidak mengambil bagian atau menarik diri jika mereka tidak menginginkannya.

Catatan Enumerator: Perkenalkan dirimu.

Nama Saya ____________________ Saat ini saya bekerja dengan tim peneliti dari Jerman dan Indonesia.

Catatan Enumerator: Jelaskan tujuan dari penelitian ini dan apa yang dibutuhkan oleh studi ini.

Kami sedang melakukan studi penelitian tentang penggunaan pendidikan sebaya untuk melengkapi perawatan diabetes yang diterima di fasilitas kesehatan masyarakat (Puskesmas) pada provinsi Aceh. Puskesmas Anda telah dipilih untuk berpartisipasi dalam penelitian ini. Ketua Puskesmas Anda [NAMA] telah setuju untuk mengambil bagian dalam penelitian dan telah memungkinkan kami untuk memilih Anda untuk melakukan wawancara singkat. [Studi ini telah disetujui oleh Pemerintah Indonesia dan didukung oleh dinas kesehatan kabupaten serta di dukung oleh Komite Etika Medis dari Universitas Goettingen di Jerman dan Komite Izin Etis Universitas Syiah Kuala.]

Catatan Enumerator: Jelaskan apa yang seharusnya dilakukan peserta jika dia memutuskan untuk ikut serta.

Kami ingin mengajukan beberapa pertanyaan tentang perawatan diabetes pada Puskesmas ini, pengetahuan Anda tentang diabetes, dan Anda dapat berbagi de tentang pendidikan/perawatan diabetes tambahan untuk pasien dengan diabetes tipe 2 di luar Puskesmas. Tidak ada jawaban yang benar atau salah; kami hanya ingin mempelajari lebih lanjut bagaimana Anda merawat orang dengan diabetes tipe 2 sebagai [dokter / perawat], karena Anda memainkan peran penting dalam praktik perawatan diabetes di fasilitas ini. Kami berencana untuk kembali ke fasilitas kesehatan Anda setelah sembilan bulan untuk menanyakan pertanyaan serupa tentang pekerjaan Anda. Setiap informasi baru yang dikembangkan selama penelitian yang dapat memengaruhi kesediaan Anda untuk melanjutkan partisipasi akan dikomunikasikan kepada Anda.

Selain Anda dan beberapa kolega Anda, kami juga akan mengajukan pertanyaan yang sama kepada tenaga kesehatan lain yang bekerja di berbagai fasilitas kesehatan di provinsi Aceh.

Catatan Enumerator: Jelaskan berapa lama wawancara dan pengamatan ini dilakukan.

Wawancara akan berlangsung sekitar 20 menit.

Catatan Enumerator: Pastikan bahwa semua informasi bersifat rahasia dan anonim.

Informasi apa pun yang Anda berikan akan dijaga kerahasiaannya. Ini berarti apa yang akan Anda katakan akan dibagikan kepada anggota tim peneliti lainnya, tetapi saya tidak akan memberi tahu kolega Anda, kepala Puskesmas Anda, atau siapa pun di komunitas Anda tentang apa yang Anda ceritakan kepada saya. Nama Anda tidak akan dicantumkan sehingga kami dapat menggambarkan apa yang Anda pikirkan tanpa ada yang tahu bahwa itu adalah Anda. Kami juga akan menyamarkan nama fasilitas kesehatan tempat Anda bekerja.

Catatan Enumerator: Jelaskan tentang prosedur pelaporan.

Hasil rekaman wawancara ini akan disimpan di komputer dan digunakan untuk menyiapkan laporan yang kami tulis setelah kami selesai melakukan wawancara dengan semua petugas kesehatan. Kami berbagi informasi yang kami kumpulkan dengan peneliti terpercaya lainnya dari Indonesia dan negara lain. Kami berharap laporan ini akan bermanfaat bagi pemerintah lokal dan nasional ketika mencoba meningkatkan perawatan diabetes pada sebuah fasilitas kesehatan di masa depan.

Catatan Enumerator: Pastikan bahwa Anda tidak meningkatkan harapan.

Penelitian kami mungkin tidak mengubah hal-hal penting dalam waktu yang cepat, karena hal itu bergantung pada pemerintah lokal dan nasional. Kami di sini untuk belajar dari Anda, tetapi kami tidak dapat berjanji untuk meningkatkan lingkungan kerja Anda.

Catatan Enumerator: Pastikan bahwa peserta memahami bahwa dia dapat berhenti kapan pun atau tidak menjawab pertanyaan apa pun pada titik mana pun.

Partisipasi dalam survei ini bersifat sukarela dan Anda dapat memilih untuk tidak menjawab beberapa pertanyaan atau semua pertanyaan. Anda berhak menolak partisipasi Anda atau berhenti berpartisipasi dalam studi ini kapan pun yang Anda mau. Anda juga bebas menjawab atau tidak menjawab pertanyaan apa pun yang Anda inginkan. Anda bebas berubah pikiran kapan saja selama perjalanan penelitian ini, tentu tanpa memengaruhi pekerjaan Anda. Namun, kami berharap Anda akan berpartisipasi dalam penelitian ini karena partisipasi Anda penting untuk membantu kami belajar tentang praktik perawatan diabetes pada fasilitas kesehatan di Aceh dan Indonesia.

Catatan Enumerator: Berikan rincian kontak peserta dari orang yang bertanggung jawab atas penelitian ini dan apabila terdapat pertanyaan dapat dikonsultasikan setelah wawancara selesai dan harap membagikan salinan lembaran wawancara yang memberikan semua informasi yang baru saja Anda tulis.

Jika Anda memiliki pertanyaan lebih lanjut, Anda dapat menghubungi:

<u>Tim Peneliti dari Jerman</u>
Till Seuring
Email: t.seuring@gmail.com
Phone: +49 (0) 421 – 21 85 69 23
Mobile: +49 (0) 176 – 47 13 41 74

<u>Tim Peneliti dari Indonesia</u>
Marthoenis
Email: marthoenis@unsyiah.ac.id
Phone: +62 (0) 812 – 63 46 34 49

Catatan Enumerator: Beri peserta kesempatan dan waktu yang cukup untuk merumuskan pertanyaan.

Apakah Anda memiliki pertanyaan pada saat ini? Anda dapat menanyakan apa pun yang tidak Anda mengerti atau apa pun yang ingin Anda ketahui.

Apakah Anda ingin berpartisipasi?

Catatan Enumerator: Jika peserta menjawab "tidak", tanyakan lagi apakah dia memiliki beberapa pertanyaan, yang membuat ia tidak berpartisipasi. Jika dia masih tidak mau berpartisipasi dan hentikanlah wawancara. Jika dia menjawab "ya", mintalah dia menandatangani formulir persetujuan.

Bolehkah saya meminta Anda untuk menandatangani formulir persetujuan ini?

LEMBAR PERSETUJUAN

KESEDIAAN MEMBERIKAN INFORMASI		
II 1	Nama Lembaga Kesehatan	
II2	Nama Tenaga Kesehatan	

Saya, ________________________________, telah membaca dan memahami formulir persetujuan, dan saya secara sukarela berpartisipasi dalam penelitian ini. Saya mengetahui bahwa saya akan menerima salinan formulir ini. Saya secara sukarela memilih untuk berpartisipasi, tetapi saya memahami bahwa persetujuan saya juga untuk tidak mengambil hak hukum apa pun dalam hal kelalaian atau kesalahan hukum lainnya dari siapa pun yang terlibat dalam penelitian ini. Saya lebih lanjut memahami bahwa tidak ada dalam bentuk persetujuan ini yang dimaksudkan untuk berlawanan dari hukum negara, atau norma lokal yang berlaku.

Nama Responden (*Cetak/Tulis tangan*):

Tanda tangan Responden:

Tanggal:

Nama Enumerator (*Cetak/Tulis tangan*):

Tanda tangan enumerator:

Tanggal:

D: FORMULIR PERSETUJUAN PASIEN (HbA1c, Kolesterol, Hemoglobin)

**Informasi Kegiatan**	
Peneliti Utama: Prof Dr Sebastian Vollmer	Institusi : Georg-August University of Goettingen
Alamat :<br><br>Georg-August Universität<br><br>Goettingen<br><br>Platz der Goettinger Sieben 3<br><br>37073 Goettingen<br><br>Jerman	Till Seuring<br><br>Phone: +49 (0) 421 – 21 85 69 23<br><br>Email: till.seuring@gmail.com<br><br>Marthoenis Marthoenis<br><br>Phone: +62 (0) 812 – 63 46 34 49<br><br>Email: marthoenis@unsyiah.ac.id
Anggota Peneliti :<br><br>Dr. Till Seurin<br><br>Dr. Marthoenis Marthoenis	Institusi:<br>Leibniz Institute for Prevention Research and Epidemiology - BIPS<br><br>nstitusi: Universitas Syiah Kuala

Catatan Penting untuk Enumerator: Tidak ada staf proyek yang harus menekan, memaksa atau menipu responden dalam upaya untuk memastikan partisipasi mereka. Staf juga harus mencoba memastikan bahwa responden tidak ditekan oleh keluarga atau anggota masyarakat lain, atau oleh staf kesehatan di fasilitas tersebut. Staf tidak boleh membuat janji apa pun yang tidak bisa atau tidak mungkin mereka tepati. Para responden akan bebas untuk menarik diri dari studi kapan saja. Sementara prosedur penelitian dirancang untuk memastikan bahwa persetujuan diinformasikan dan bersifat sukarela, satu-satunya orang yang benar-benar dapat memastikannya adalah Enumerator. Anda harus berusaha semaksimal mungkin untuk memastikan peserta memahami tujuan penelitan dan merasa bebas untuk tidak mengambil bagian atau menarik diri jika mereka tidak menginginkannya.

Catatan Enumerator: Perkenalkan dirimu.

Nama saya ___________________ Saat ini saya bekerja dengan tim peneliti dari Jerman dan Indonesia.

Catatan Enumerator: Jelaskan tujuan dari penelitian dan apa yang dipelajari oleh studi ini.

Kami sedang melakukan studi penelitian tentang penggunaan pendidikan sebaya untuk membantu orang dengan diabetes tipe 2 yang menerima perawatan mereka di Puskesmas pada provinsi Aceh. Karena Anda telah menjadi pasien di salah satu Puskesmas yang menjalin kerjasama dengan kami, Anda telah dipilih untuk berpartisipasi dalam survei ini. [Studi ini telah disetujui dan didukung oleh Pemerintah Indonesia dan Komite Etika Medis dari Universitas Goettingen di Jerman serta Komite Izin Etis di Fakultas Kedokteran Universitas Syiah Kuala.]

Catatan Enumerator: Jelaskan apa yang seharusnya dilakukan peserta jika dia memutuskan untuk berpartisipasi.

Sebelum memulai dengan program pendidikan sebaya, kami ingin menanyakan beberapa pertanyaan tentang latar belakang Anda, kesehatan Anda, dan penyakit diabetes tipe 2 Anda. Ini akan mencakup usia Anda, pendidikan, tahun Anda diagnosis diabetes tipe 2, pengobatan diabetes, kesehatan Anda secara keseluruhan dan setiap penyakit lain yang mungkin Anda miliki. Selain itu, ini akan mencakup pertanyaan tentang penggunaan perawatan kesehatan Anda dan setiap biaya yang mungkin Anda keluarkan terkait dengan perawatan diabetes.

Selain menjawab kuesioner, bentuk partisipasi Anda dalam penelitian ini juga mencakup pengukuran kadar hemoglobin terglikosimu (HbA1c) sehingga kita dapat mengetahui seberapa efektif intervensi pendidikan sebaya ini (*peer education*). HbA1c menunjukkan kadar glukosa darah rata-rata Anda selama dua hingga tiga bulan terakhir. Risiko utama diabetes adalah kadar glukosa darah yang tinggi karena merusak arteri dan organ Anda dan menyebabkan masalah kesehatan yang sangat parah dari waktu ke waktu, seperti kebutaan, penyakit jantung atau gagal ginjal. Salah satu tujuan utama dari penelitian ini adalah untuk membantu Anda mencegah kadar glukosa darah yang tinggi. Oleh karena itu sangat penting bagi kami untuk mengetahui tingkat HbA1c Anda sebelum dimulainya intervensi pendidikan sebaya dan setelah dimulai nanti. Keuntungan bagi Anda dari tes ini adalah Anda akan menerima informasi tentang kadar glukosa darah Anda saat ini yang dapat Anda gunakan pada saat konsultasi dengan dokter Anda berikutnya mengenai penyakit diabetes Anda.

Selanjuntya kami juga ingin melakukan pengecekan terhadap kadar kolesterol dan hemoglobin Anda. Kadar kolesterol yang tinggi akan menyebabkan tingginya risiko terkena

penyakit jantung. Kami juga ingin mengetahui penelitian ini apakah dapat mengurangi kadar kolesterol Anda. Kadar hemoglobin penting karena dapat memberi informasi kepada Anda dan tim peneliti tentang kemungkinannya Anda kekurangan zat besi dalam darah Anda, gejala yang ditimbulkan seperti kelelahan dan sakit kepala, dan juga dapat menyebabkan masalah jantung. Lebih jauh, keuntungan bagi Anda dari tes yang dilakukan ini bahwa Anda akan menerima informasi tentang kadar kolesterol dan hemoglobin Anda saat ini yang dapat digunakan pada saat konsultasi ke dokter Anda.

Orang-orang terlatih akan melakukan pengecekan darah Anda. Mereka akan menggunakan jarum untuk mengambil sejumlah kecil darah dari lengan Anda dan menganalisisnya dengan perangkat canggih kecil di hadapan Anda. Selanjutnya, Anda akan menerima hasil tes segera setelah dinyatakan selesai. Proses ini mungkin sedikit sakit untuk sedetik, namun Anda dapat percaya pada rekan saya yang terlatih dan yang sangat berpengalaman dalam mengambil sampel darah. Keputusan memberikan izin untuk pengambilan darah ini sepenuhnya ada pada Anda, namun Anda tidak akan dapat mengambil bagian dalam studi jika Anda menolak untuk melakukan tes darah Anda. Harap diketahui bahwa HbA1c, kolesterol dan hemoglobin Anda adalah data kesehatan yang sangat sensitif.

Selain Anda, kami juga akan mengajukan pertanyaan yang sama dan mengambil tes darah dari pasien lain dari ini dan Puskesmas lainnya yang merupakan bagian dari penelitian kami.

Catatan Enumerator: Jelaskan berapa lama wawancara dan penyelidikan dilakukan.

Wawancara akan berlangsung selama sekitar 45 menit.

Catatan Enumerator: Pastikan bahwa semua informasi bersifat rahasia dan anonim.

Informasi apa pun yang Anda berikan akan dijaga kerahasiaannya hanya kepada anggota tim peneliti lainnya, tetapi saya tidak akan memberi tahu dokter, keluarga, atau siapa pun di komunitas Anda apa yang Anda katakan kepada saya. Nama Anda tidak akan dicantumkan sehingga kami dapat menggambarkan apa yang Anda pikirkan tanpa ada yang tahu bahwa itu adalah Anda. Hasil HbA1c Anda akan disimpan secara terpisah dari nama Anda.

Catatan Enumerator: Jelaskan tentang prosedur pengarsipan.

Rekaman wawancara akan disimpan di komputer dan digunakan untuk menyiapkan laporan yang kami tulis setelah kami selesai melakukan wawancara dengan pasien. Kami berbagi informasi yang kami kumpulkan dengan peneliti terpercaya lainnya dari Indonesia dan negara lain. Kami berharap laporan ini akan berguna bagi pemerintah lokal dan nasional ketika merencanakan perawatan diabetes di masa depan.

Catatan Enumerator: Pastikan bahwa Anda tidak meningkatkan harapan.

Penelitian kami mungkin tidak mengubah kesehatan Anda dalam waktu cepat, karena itu tergantung pada keberhasilan program pendidikan sebaya yang ingin kami selidiki. Kami di sini untuk belajar dari Anda dan pengalaman Anda dengan diabetes, tetapi kami tidak dapat berjanji untuk meningkatkan kehidupan Anda atau keluarga Anda.

Catatan Enumerator: Berikan rincian kontak peserta dari orang yang bertanggung jawab atas proyek yang dapat dikonsultasikan jika pertanyaan muncul setelah wawancara selesai dan membagikan selembar kertas yang memberikan semua informasi yang baru saja Anda tuliskan.

Jika Anda memiliki pertanyaan lebih lanjut, Anda dapat menghubungi:

Tim Peneliti dari Jerman
Till Seuring
Email: t.seuring@gmail.com
Phone: +49 (0) 421 – 21 85 69 23
Mobile: +49 (0) 176 – 47 13 41 74

Tim Peneliti dari Indonesia
Marthoenis
Email: marthoenis@unsyiah.ac.id
Phone: +62 (0) 812 – 63 46 34 49

Catatan Enumerator: Beri peserta kesempatan dan waktu yang cukup untuk merumuskan pertanyaan.

Apakah Anda memiliki pertanyaan pada saat ini? Anda dapat menanyakan apa pun yang tidak Anda mengerti atau apa pun yang ingin Anda ketahui.

Apakah Anda ingin berpartisipasi?

Catatan Enumerator: Jika peserta menjawab "tidak", tanyakan lagi apakah dia memiliki beberapa pertanyaan, yang mencegah dia untuk berpartisipasi. Jika dia masih tidak mau berpartisipasi maka hentikanlah wawancara. Jika dia menjawab "ya", mintalah dia menandatangani formulir persetujuan.

Bolehkah saya meminta Anda untuk menandatangani formulir persetujuan ini?

LEMBAR PERSETUJUAN

**KESEDIAAN MEMBERIKAN INFORMASI**		
**II1**	Nama Lembaga Kesehatan	
**II2**	Nama Pasien:	Tanggal lahir pasien:

Saya, ________________________________, telah membaca dan memahami formulir persetujuan, dan saya secara sukarela berpartisipasi dalam penelitian ini. Saya mengerti bahwa saya akan menerima salinan formulir ini. Saya secara sukarela memilih untuk berpartisipasi, tetapi saya memahami bahwa persetujuan saya juga termasuk tidak mengambil hak hukum apa pun dalam hal kelalaian atau kesalahan hukum lainnya dari siapa pun yang terlibat dalam penelitian ini. Saya lebih lanjut memahami bahwa tidak ada dalam bentuk persetujuan ini yang dimaksudkan berlawanan dengan hokum negara dan norma sosial di daerah yang berlaku.

Nama Responden (*Cetak/Tulis tangan*):

Tanda tangan Responden:

Tanggal:

Nama Enumerator (*Cetak/Tulis tangan*):

Tanda tangan Enumerator:

Tanggal:
