# Supplementary material for: Using peer education to improve diabetes management and outcomes in a low-income setting: a randomized controlled trial
Source: Trials. 2019 Sep 2;20:548. doi: 10.1186/s13063-019-3656-1 (PMC6719346; doi:10.1186/s13063-019-3656-1)
Supplement: Supplementary file 3 — Consent Form (patients and facilities) in English. (PDF 313 kb) [file 13063_2019_3656_MOESM3_ESM.pdf]

A: HEALTH INSTITUTION CONSENT FORM

**Project Information**	
Principal Investigator: Prof. Dr.<br>Sebastian Vollmer	Organization: Georg-August University of Goettingen
Location:<br>Georg-August Universität<br>Goettingen<br>Platz der Goettinger Sieben 3<br>37073 Goettingen<br>Germany	Till Seuring<br>Phone: +49 (0) 421 – 21 85 69 23<br>Email: till.seuring@gmail.com<br>Marthoenis Marthoenis<br>Phone: +62 (0) 812 – 63 46 34 49<br>Email: marthoenis@unsyiah.ac.id
Other Investigators:<br>Dr. Till Seuring<br>Dr. Marthoenis	Organization:<br>Leibniz Institute for Prevention Research and Epidemiology - BIPS<br>Syiah Kuala University

My name is ___________________ Currently I am working with a team from the University in Goettingen, Germany and Syiah Kuala University, Banda Aceh. We are conducting an intervention about the use of peer education for people with diabetes to compliment diabetes care at Puskesmas. Although the research we conduct is independent from the government, the district health offices of Aceh support its introduction and the Ethical Clearance Committee at the Medical Faculty of Syiah Kuala University has given approval to conduct this study [show support letters].

Peer education is an intervention that aims to increase the knowledge about diabetes self-care and healthy behaviours of patients with type 2 diabetes, in order to improve their blood glucose levels and reduce diabetes risk factors such as obesity, overweight and high blood pressure. Peer education has been shown to achieve these goals in other countries, so that it could help to reduce the burden of diabetes in Aceh as well. To carry out this project we will select patients with type 2 diabetes as well as peer educators, i.e. people with diabetes that will receive additional training in diabetes education. These peer educators will then lead groups of people with type 2 diabetes and educate them about how to better treat their diabetes and what to do to change their lifestyle.

The aim of the study is that in the end, peer education groups will be formed at all participating public health facilities. But due to budget constraints we cannot introduce the groups together with a complementary training in all facilities at the same time. Hence, we will start to implement it using patients from 50% of all health facilities and the other half of the facilities will get it by the end of 2020. It will be determined by a lottery which institution will get the intervention first. Right now we are visiting all health facilities to ask their consent to take part in the research study. Shortly, a person will come back to your health institution and tell you if your facility got selected for the implementation by the middle of 2019 or by the end of 2020.

Our research may not change things in the short term, because that depends on local and national governments. We are here to learn from you and your patients, but we cannot promise to improve things. Participation in this research study is voluntary. However, we hope that you will participate in this survey since your participation is important to help us learn about how to reduce the burden of diabetes in your province and throughout Indonesia.

In case you have any further questions, when I have already left, you can contact:

German research team

Till Seuring

Email: t.seuring@gmail.com

Phone: +49 (0) 421 – 21 85 69 23

Mobile: +49 (0) 176 – 47 13 41 74

Indonesian research team

Marthoenis Marthoenis

Phone: +62 (0) 812 – 63 46 34 49

Email: marthoenis@unsyiah.ac.id

Do you have any question at this point? You can ask me anything that you did not understand or anything you want to know.

Would you like to participate?

CONSENT FORM

**IDENTIFICATION INFORMATION**		
II 1	Health Facility Name	Name:

I, ________________________________, after being informed about all aspects of this project described in this format, and having all my questions and concerns about this project answered, I voluntarily accept to participate this project in my health facility. I commit myself and my health facility to support the procedures described above. I have had the opportunity to ask any questions related to the project. I understand the procedures of the project and how the information will be treated in a confidential manner, without revealing the identity of any person participating in the project in any result reported or published. I give my authorization to give access to this information to all members of the research team, knowing that this information will be used confidentially. I understand that my consent does not take away any legal rights in the case of negligence or other legal fault of anyone who is involved in this study. I further understand that nothing in this consent form is intended to replace any applicable federal, state, or local laws.

Principal Name (Printed or Typed):

Principal Signature:

Date:

Field Worker Name (Printed or Typed):

Signature of Field Worker:

Date:

B: HEALTH PERSONNEL CONSENT FORM

**Project Information**	
Principal Investigator: Prof. Dr.<br>Sebastian Vollmer	Organization: Georg-August University of Goettingen
Location:<br>Georg-August Universität<br>Goettingen<br>Platz der Goettinger Sieben 3<br>37073 Goettingen<br>Germany	Till Seuring<br>Phone: +49 (0) 421 – 21 85 69 23<br>Email: till.seuring@gmail.com<br>Marthoenis Marthoenis<br>Phone: +62 (0) 812 – 63 46 34 49<br>Email: marthoenis@unsyiah.ac.id
Other Investigators:<br>Dr. Till Seuring<br>Dr.Marthoenis Marthoenis	Organization:<br>Leibniz Institute for Prevention Research and Epidemiology - BIPS<br>Syiah Kuala University

My name is ___________________ Currently I am working with a team of researchers from Germany and Indonesia.

We are conducting a research study about the use of peer education to complement diabetes care received at public health facilities in Aceh province. Your health facility has been chosen to participate in the study. The principal of your health facility has agreed to take part in the study and has allowed us to select you for a short questionnaire. [The study has been approved by the Government of Indonesia and is supported by the district health office. The Medical Ethics Committee from the University of Goettingen in Germany and the Ethical Clearance Committee of Syiah Kuala University approved the study.]

We would like to ask you some questions about diabetes care here at the health facility, your knowledge about diabetes, and if you think additional diabetes education for patients with type 2 diabetes outside the health facility would be worthwhile. There are no right or wrong answers; we just want to learn more how you care for people with type 2 diabetes as a [doctor/nurse], as you play an important role in the diabetes care practices in this facility. We are planning to come back to your health facility after nine months to ask you

similar questions concerning your work. Any new information developed during the study that may affect your willingness to continue participation will be communicated to you.

Apart from you and some of your colleagues, we are also going to ask the same questions to other health personnel working in different health facilities in the province of Aceh.

The interview will last for approximately 20 minutes.

Whatever information you provide will be kept strictly confidential. This means what you will say will be shared with other members of the research team, but I am not going to tell your colleagues, your principal, or anybody in the community what you tell me. Your name will not be used so we can describe what you think without anyone knowing that it is you. We will also disguise the name of the health facility you are working in.

Bits of what you say will be stored on a computer and used to prepare a report that we write after we have talked to all the health personnel. We are sharing the information that we collect with other trusted researchers from Indonesia and other countries. We hope this report will be helpful to local and national governments when trying to improve diabetes care in health facilities in the future.

Our research may not change things in the short term, because that depends on local and national governments. We are here to learn from you, but we cannot promise to improve your working environment.

Participation in this survey is voluntary and you can choose not to answer any question or all of the questions. You have the right to reject your participation or to stop participating in this study at any time that you want. You are also free to answer or not to any questions that you want. You are free to change your mind at any time during this project, without affecting your job. However, we hope that you will participate in this survey since your participation is important to help us learn about diabetes care practices in health facilities in Aceh and Indonesia.

In case you have any further questions, when I have already left, you can contact:

German research team

Till Seuring

Email: t.seuring@gmail.com

Phone: +49 (0) 421 – 21 85 69 23

Mobile: +49 (0) 176 – 47 13 41 74

Indonesian research team

Marthoenis Marthoenis

Phone: +62 (0) 812 – 63 46 34 49

Email: marthoenis@unsyiah.ac.id

Do you have any question at this point? You can ask me anything that you did not understand or anything you want to know.

Would you like to participate?

May I ask you to sign this consent form?

CONSENT FORM

II 1	Health Facility Name
II2	Health Personnel Name

I, _________________________________, have read and understood the consent form, and I volunteer to participate in this research study. I understand that I will receive a copy of this form. I voluntarily choose to participate, but I understand that my consent does not take away any legal rights in the case of negligence or other legal fault of anyone who is involved in this study. I further understand that nothing in this consent form is intended to replace any applicable Federal, state, or local laws.

Participant Name (Printed or Typed):

Participant Signature:

Date:

Field Worker Name (Printed or Typed):

Signature of Field Worker:

Date:

D: PATIENT CONSENT FORM (HbA1c, cholesterol, hemoglobin)

Project Information	
Principal Investigator: Prof. Dr. Sebastian Vollmer	Organization: Georg-August University of Goettingen
Location:<br>Georg-August Universität<br>Goettingen<br>Platz der Goettinger Sieben 3<br>37073 Goettingen<br>Germany	Till Seuring<br>Phone: +49 (0) 421 – 21 85 69 23<br>Email: till.seuring@gmail.com<br>Marthoenis Marthoenis<br>Phone: +62 (0) 812 – 63 46 34 49<br>Email: marthoenis@unsyiah.ac.id
Other Investigators:<br>Dr. Till Seuring<br>Dr. Marthoenis	Organization:<br>Leibniz Institute for Prevention Research and Epidemiology - BIPS<br>Syiah Kuala University

My name is ____________________ . Currently I am working with a team of researchers from Germany and Indonesia.

We are conducting a research study about the use of peer education to help people with type 2 diabetes that receive their care at public health institutions in Aceh province. As you have been a patient in one of our included health facilities, you have been chosen to participate in this survey. [The study has been approved and is supported by the Government of Indonesia and by the Medical Ethics Committee from the University of Goettingen in Germany and the Ethical Clearance Committee at the Medical Faculty of Syiah Kuala University.]

Before we start with the peer education program, we would like to ask you some questions about your background, your health and your type 2 diabetes. This will include your age, education, your year of diagnosis of type 2 diabetes, the treatment of diabetes, your overall health and any other diseases you may have. Additionally, this will include questions about your healthcare usage and any expenditures you may have had related to the treatment of diabetes.

Apart from answering the questionnaire, participation in this study also includes a

measurement of your glycated hemoglobin (HbA1c) levels so that we can know how effective the peer education intervention is. HbA1c shows you and us your average blood glucose levels over the last two to three months. The main risk of diabetes is that high blood glucose levels damage your arteries and organs and lead to very severe health problems over time, such as blindness, heart disease or renal failure. One of the main goals of this study is to help you to prevent these high blood glucose levels. It is therefore very important for us to know your HbA1c levels before the start of the peer education intervention and after its start. The advantage for you from this test is that you will receive information about your current blood glucose levels that you can use at your next physician appointment to discuss the further treatment of your diabetes.

Further, we would like to measure your cholesterol levels and hemoglobin levels. High cholesterol levels can be a risk factor for heart disease. We would therefore like to know, if this study also helps to reduce cholesterol levels in people with diabetes. Hemoglobin levels are important as they can tell you and us about a potential iron deficiency in your blood which can have symptoms such as tiredness and headaches, and can lead to heart problems. Again, the advantage for you from these tests is that you will receive information about your current cholesterol and hemoglobin levels that you can use at your next physician appointment.

Local trained people are going to perform the blood tests. They will take a few small drops of blood from your fingertip and analyze them with a small handheld device in your presence. You will receive the test results immediately after the tests concluded. The blood draw might hurt a little bit for a second as we will need to prick your fingertip, however, the people performing the test have been trained to perform these tests to minimize any discomfort to you. The decision of giving the permission to the blood testing is fully optional for you, however you will not be able to take part in the study if you decline to have your blood tested. Please be aware that your HbA1c, cholesterol and hemoglobin levels are very sensitive health data.

Apart from you, we are also going to ask the same questions to and take a blood test from the other patients from this and other health facilities that are part of our study.

The interview will last for approximately 45 minutes.

Whatever information you provide will be kept strictly confidential. This means what you will say will be shared with other members of the research team, but I am not going to tell your doctor, family or anyone in your community what you tell me. Your name will not be

used so we can describe what you think without anyone knowing that it is you. Your blood test results will be stored separately from your name.

Bits of what you say will be stored on a computer and used to prepare a report that we write after we have talked to the patients. We are sharing the information that we collect with other trusted researchers from Indonesia and other countries. We hope this report will be helpful to local and national governments when planning diabetes care in the future.

Our research may not change your health in the short term, because that depends on the success of the peer education program which we want to investigate. We are here to learn from you and your experience with diabetes, but we cannot promise to improve your or your family's life.

In case you have any further questions when I have already left you can contact:

German research team
Till Seuring

Email: t.seuring@gmail.com

Phone: +49 (0) 421 – 21 85 69 23

Mobile: +49 (0) 176 – 47 13 41 74

Indonesian research team

Marthoenis Marthoenis

Phone: +62 (0) 812 – 63 46 34 49

Email: marthoenis@unsyiah.ac.id

Do you have any question at this point? You can ask me anything that you did not understand or anything you want to know.

Would you like to participate?

May I ask you to sign this consent form?

CONSENT FORM

IDENTIFICATION INFORMATION		
**II1**	Health Facility Name	
**II2**	Patient Name, Date of birth	Name

I, _________________________________, have read and understood the consent form, and I volunteer to participate in this research study. I understand that I will receive a copy of this form. I voluntarily choose to participate, but I understand that my consent does not take away any legal rights in the case of negligence or other legal fault of anyone who is involved in this study. I further understand that nothing in this consent form is intended to replace any applicable Federal, state, or local laws.

Participant Name (Printed or Typed):

Participant Signature:

Date:

I, _________________________________, have read all the information to the participant.

Field Worker Name (Printed or Typed):

Signature of Field Worker:

Date:
